# Efficacy and safety of immune checkpoint inhibitors plus chemotherapy in early-stage triple-negative breast cancer: a systematic review and meta-analysis

**DOI:** 10.3389/fonc.2026.1853057

**Published:** 2026-09-24

**Authors:** Jia-Le Li, Pei-Tao Wu, Jing-Shuang Song, Hua-Wei Yang

**Affiliations:** 1 Department of Breast Surgery, Guangxi Medical University Cancer Hospital, Nanning, Guangxi, China; 2 Department of Breast and Thyroid Surgery, The First Affiliated Hospital of Guilin Medical University, Guilin, Guangxi, China; 3 Guilin Medical University, Guilin, Guangxi, China

**Keywords:** adverse events, chemotherapy, immunotherapy, meta-analysis, pathological complete response, systematic review, triple-negative breast cancer

## Abstract

**Background:**

Immune checkpoint inhibitors combined with chemotherapy have improved pathological responses in early-stage triple-negative breast cancer (TNBC), but the magnitude of benefit and associated toxicity remain variable across trials. This systematic review and meta-analysis aimed to evaluate the efficacy and safety of immunotherapy plus chemotherapy in patients with early-stage or high-risk TNBC.

**Methods:**

This study was conducted in accordance with the Preferred Reporting Items for Systematic Reviews and Meta-Analyses (PRISMA) statement. PubMed, Embase, Web of Science, and the Cochrane Library were searched from inception to March 2026 for randomized controlled trials (RCTs). The primary outcome was pathological complete response (pCR). Dichotomous outcomes were pooled as risk ratios (RRs) with 95% confidence intervals (CIs). Between-study heterogeneity was assessed using Cochran’s Q test and the I² statistic. Exploratory subgroup analyses were conducted according to programmed death-ligand 1 (PD-L1) expression and lymph node status.

**Results:**

Five RCTs were included. Immunotherapy plus chemotherapy was associated with a higher pCR rate than control regimens (RR = 1.44, 95% CI 1.09–1.79), with substantial heterogeneity (I² = 83.7%). In exploratory analyses, the pooled RR was 1.36 (95% CI 1.18–1.57) in PD-L1-positive patients and 1.18 (95% CI 0.96–1.45) in PD-L1-negative patients. Corresponding RRs were 1.42 (95% CI 1.19–1.69) for node-positive and 1.21 (95% CI 1.02–1.43) for node-negative disease. Immunotherapy-containing regimens were also associated with higher risks of grade 3–4 adverse events (RR = 1.18, 95% CI 1.05–1.33), serious adverse events (RR = 1.42, 95% CI 1.12–1.79), and treatment discontinuation due to adverse events (RR = 1.67, 95% CI 1.18–2.36).

**Conclusions:**

Immunotherapy combined with chemotherapy was associated with higher pCR rates in early-stage or high-risk TNBC but also with greater treatment-related toxicity. PD-L1 and nodal subgroup findings should be considered exploratory, and further evidence is needed to clarify long-term clinical outcomes.

**Systematic review registration:**

[url], identifier [].

## 1 Introduction

Triple-negative breast cancer (TNBC), defined by the absence of estrogen receptor, progesterone receptor, and human epidermal growth factor receptor 2 expression or amplification, accounts for approximately 15%–20% of breast cancers (1). Compared with other breast cancer subtypes, TNBC is characterized by higher histological grade, rapid proliferation, early recurrence, and increased risk of distant metastasis (2). Although early-stage TNBC is potentially curable, long-term outcomes remain unsatisfactory in patients with residual invasive disease after neoadjuvant therapy (3, 4). Neoadjuvant chemotherapy has long been a standard strategy for high-risk early-stage TNBC because it can reduce tumor burden, facilitate breast-conserving surgery, and provide an *in vivo* assessment of treatment sensitivity (5). Pathological complete response (pCR) is widely used as a clinically relevant endpoint, as patients achieving pCR generally have more favorable outcomes than those with residual disease (6, 7). However, because pCR is an imperfect trial-level surrogate for long-term survival, it should be interpreted together with mature survival outcomes when evaluating emerging therapeutic strategies (8, 9).

Immune checkpoint inhibitors have reshaped the treatment landscape of TNBC. The immunogenic features of TNBC, including increased tumor-infiltrating lymphocytes, greater genomic instability, and activation of immune-related pathways, provide a rationale for combining immunotherapy with cytotoxic chemotherapy (10, 11). Chemotherapy may further enhance antitumor immunity by promoting immunogenic cell death, increasing antigen release, and modifying the tumor microenvironment, thereby potentially augmenting programmed death 1/programmed death-ligand 1 (PD-1/PD-L1) blockade. Randomized trials have evaluated pembrolizumab, atezolizumab, and durvalumab combined with chemotherapy in early-stage TNBC, mainly in the neoadjuvant setting, and several have reported improved pCR rates (12). Longer-term evidence, including the final overall survival analysis of KEYNOTE-522, has further supported the clinical relevance of this approach. Recent systematic reviews have also shown that neoadjuvant immune checkpoint inhibitors improve pCR in TNBC, although with increased toxicity (13, 14). These findings support the integration of chemoimmunotherapy into selected early-stage TNBC treatment strategies, while underscoring the need for a balanced assessment of efficacy, safety, biomarker heterogeneity, and long-term outcomes (15, 16).

Despite these advances, several uncertainties remain. Treatment effects have varied across trials, potentially reflecting differences in trial design, chemotherapy backbone, immunotherapy schedule, patient risk profile, and pCR definitions. The predictive value of PD-L1 in early-stage TNBC also remains uncertain because assays, scoring systems, and positivity cutoffs differ across studies (17, 18). Moreover, improvements in pathological response must be weighed against treatment-related toxicity and interpreted in the context of evolving long-term outcome data. Compared with previous reviews focusing primarily on overall efficacy, the present analysis further integrates trial-specific pCR definitions, PD-L1 assessment methods, clinically relevant safety outcomes, and available long-term efficacy data. Therefore, this systematic review and meta-analysis aimed to synthesize randomized evidence on the efficacy and safety of chemoimmunotherapy in early-stage or high-risk TNBC, with particular attention to pCR, clinically relevant heterogeneity, exploratory biomarker and nodal subgroups, safety outcomes, and available long-term efficacy data.

## 2 Methods

### 2.1 Search strategy

This systematic review and meta-analysis was conducted in accordance with the Preferred Reporting Items for Systematic Reviews and Meta-Analyses (PRISMA) statement (19). PubMed, Embase, Web of Science, and the Cochrane Library were systematically searched from inception to March 2026 for randomized controlled trials evaluating immune checkpoint inhibitors combined with chemotherapy for early-stage or high-risk TNBC. No language restrictions were applied. The search strategy combined controlled vocabulary and free-text terms related to TNBC, early-stage or neoadjuvant treatment, immune checkpoint inhibitors, chemotherapy, and randomized controlled trials. Detailed search strategies are provided in **Supplementary Table 1**. Reference lists of eligible studies and relevant reviews were manually screened for additional records. All retrieved citations were imported into EndNote for record management and duplicate detection. After duplicate removal, titles and abstracts were independently screened by two reviewers according to predefined eligibility criteria. Potentially eligible reports subsequently underwent independent full-text assessment. Records identified as clearly ineligible during record organization were manually verified before exclusion. Disagreements at any stage were resolved through discussion or consultation with a third reviewer.

### 2.2 Inclusion criteria and exclusion criteria

Eligibility criteria were defined according to the PICO framework. The population comprised patients with early-stage or high-risk TNBC. Trials enrolling broader breast cancer populations were eligible only when TNBC-specific efficacy data could be reliably extracted. Eligible interventions consisted of an immune checkpoint inhibitor combined with chemotherapy, with chemotherapy alone, placebo plus chemotherapy, or another non-immunotherapy regimen serving as the comparator. Studies were required to report at least one prespecified efficacy or safety outcome, including pCR, event-free survival (EFS), disease-free survival (DFS), invasive disease-free survival (iDFS), distant disease-free survival (DDFS), overall survival (OS), grade 3–4 adverse events (AEs), serious adverse events (SAEs), treatment discontinuation due to AEs, or immune-related or immune-mediated adverse events. Only randomized controlled trials with sufficient extractable data were included. Non-randomized studies, single-arm studies, reviews, case reports, conference abstracts, editorials, letters, preclinical studies, and reports without sufficient extractable data were excluded. When multiple publications reported results from the same trial, reports were collated at the trial level and the most relevant or complete report for each outcome was used to avoid double counting.

### 2.3 Data extraction

Data were independently extracted by two reviewers using a standardized data collection form. Extracted study characteristics included author, publication year, study setting, trial phase and design, analysis population, clinical stage, nodal status, PD-L1 assessment, chemotherapy backbone, immune checkpoint inhibitor and drug class, treatment timing and duration, use of postoperative immunotherapy, and follow-up duration. Discrepancies were resolved through discussion or consultation with a third reviewer.

For the primary pCR outcome, the exact trial-specific definition was recorded, including whether residual ductal carcinoma *in situ* was permitted and whether absence of invasive disease in regional lymph nodes was required. The corresponding analysis population was also recorded as intention-to-treat, modified intention-to-treat, randomized, or evaluable, as applicable. For I-SPY2, the observed TNBC-specific arm-level pCR events were used in the event/total meta-analysis, whereas the Bayesian model-estimated pCR rates reported by the adaptive trial were retained for descriptive purposes only. Missing or unclear endpoint information was recorded as not reported and was not imputed.

For PD-L1 analyses, the assay or platform, scoring system, positivity cutoff, and available subgroup pCR data were extracted. Because PD-L1 assessment differed across trials, including immune-cell and combined positive scoring systems, subgroup analyses according to PD-L1 status were considered exploratory. Nodal-status subgroup data were extracted when available. Immune checkpoint inhibitor class was also recorded; however, comparisons between anti-PD-1 and anti-PD-L1 agents were descriptive only, and no formal between-class interaction analysis was performed because of the limited number of trials and substantial clinical and methodological heterogeneity.

For safety outcomes, trial-specific safety denominators and arm-level event counts were extracted for grade 3–4 AEs, SAEs, and treatment discontinuation due to AEs. Frequently reported or clinically relevant grade 3–4 toxicities and the reported definition of AE-related treatment discontinuation were also recorded when available. Safety data were quantitatively pooled only when they were reported for a TNBC-specific or otherwise directly applicable study population. Accordingly, I-SPY2 was not included in the pooled TNBC safety analyses because its safety outcomes were primarily reported for the broader ERBB2-negative population.

Immune-related AEs, immune-mediated AEs, and adverse events of special interest were extracted separately and retained using the terminology reported in each trial. Because definitions, denominators, and reporting formats were not sufficiently comparable across trials, these outcomes were summarized descriptively rather than quantitatively pooled.

Long-term efficacy outcomes, including EFS, DFS, iDFS, DDFS, and OS, were extracted when available. Because the reported endpoints, follow-up durations, data maturity, and availability of hazard ratios with confidence intervals differed substantially across trials, long-term outcomes were summarized descriptively and were not combined in a quantitative meta-analysis.

### 2.4 Risk-of-bias assessment

The risk of bias of the included randomized controlled trials was independently assessed by two reviewers using the original Cochrane Collaboration risk-of-bias tool (20). Seven domains were evaluated: random sequence generation, allocation concealment, blinding of participants and personnel, blinding of outcome assessment, incomplete outcome data, selective reporting, and other potential sources of bias. Each domain was classified as low, high, or unclear risk of bias according to the RoB 1 framework. Disagreements were resolved through discussion or consultation with a third reviewer. Domain-level assessments were used rather than an overall numerical quality score.

### 2.5 Statistical analyses

All statistical analyses were performed using Stata version 18.0 (StataCorp LLC, College Station, TX, USA). Dichotomous outcomes were summarized as risk ratios (RRs) with 95% confidence intervals (CIs). The primary meta-analysis of pCR was based on observed arm-level event counts and denominators. For I-SPY2, observed TNBC-specific pCR data were used in the quantitative synthesis, whereas the Bayesian model-estimated rates were retained for descriptive reporting only. Between-study heterogeneity was assessed using Cochran’s Q test and the I² statistic. A random-effects model was used for the primary pCR analysis. Exploratory subgroup analyses according to PD-L1 expression and lymph node status were also performed using random-effects models. Comparisons between anti-PD-1 and anti-PD-L1 inhibitors were descriptive, and no formal between-class interaction test was performed. For safety outcomes, grade 3–4 adverse events were pooled using a random-effects model, whereas serious adverse events and treatment discontinuation due to adverse events were analyzed using fixed-effect models. Leave-one-out sensitivity analysis was performed for the primary pCR outcome by sequentially excluding each study and recalculating the pooled estimate. Immune-related adverse events and long-term efficacy outcomes were summarized descriptively because their definitions, reporting formats, endpoints, and follow-up periods were not sufficiently comparable for quantitative pooling. Formal assessment of publication bias was not performed because only five trials contributed to the primary meta-analysis.

## 3 Results

### 3.1 Search results and study selection

A total of 527 records were identified through database and register searches, including 501 records from databases and 26 from registers. Before screening, 185 duplicate records were removed using EndNote reference-management software. In addition, 106 records identified as clearly ineligible during initial record organization and 98 records removed for other reasons were manually verified before exclusion. As a result, 138 records remained for title and abstract screening. After screening, 118 records were excluded, and 20 reports were sought for retrieval. Of these, 2 reports could not be retrieved, leaving 18 reports for full-text eligibility assessment. Following full-text review, 13 reports were excluded for the following reasons: reviews (n=6), sequentially published articles (n=2), insufficient data for extraction (n=3), and clinical trials without control groups (n=2). Ultimately, 5 studies met the predefined eligibility criteria and were included in the systematic review and meta-analysis (**Figure 1**).

**Figure 1:**
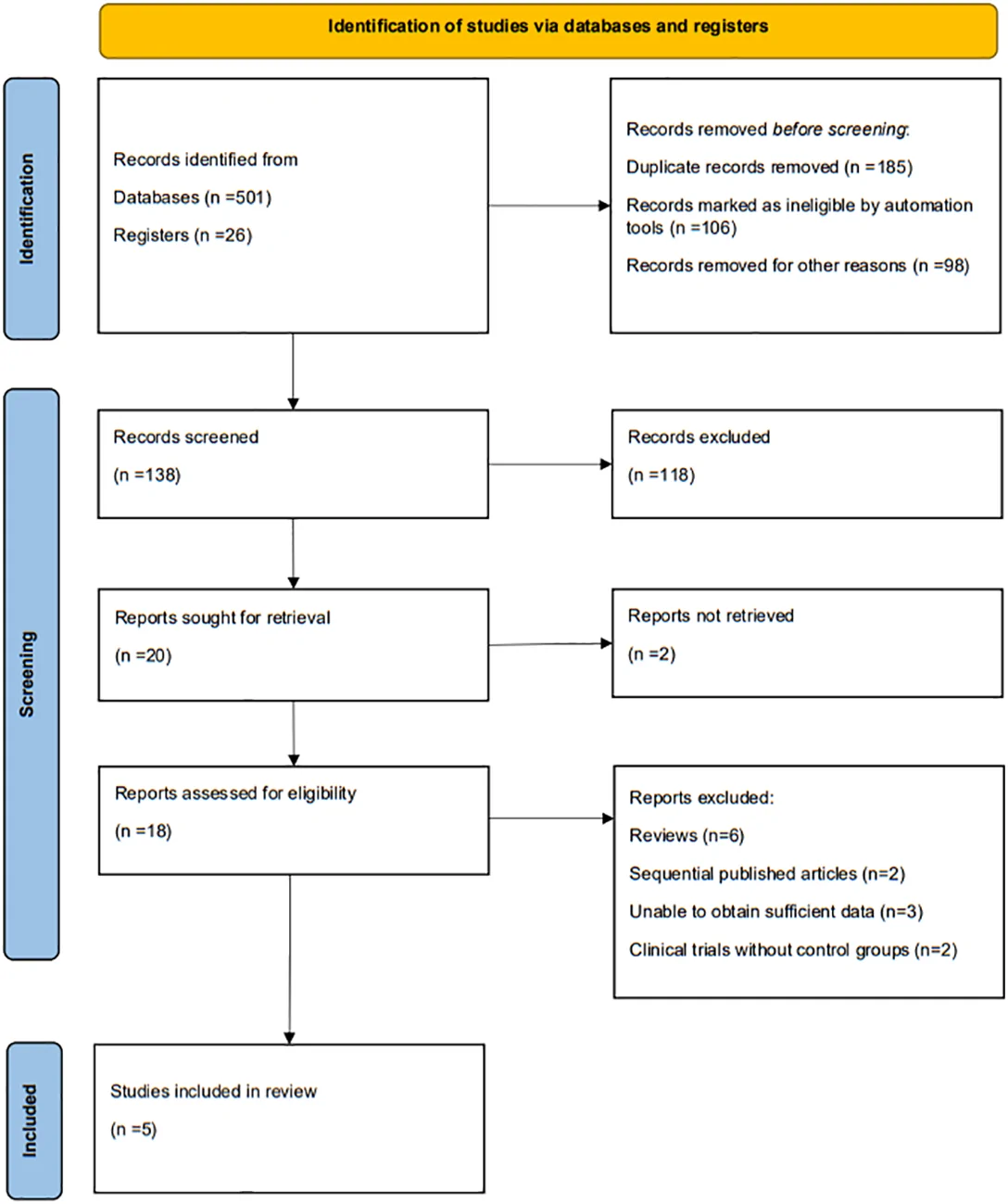
Flow diagram of the study selection process for the systematic review and meta-analysis.

### 3.2 Study characteristics

Five randomized clinical trials were included in the systematic review and meta-analysis. The study populations primarily comprised patients with early-stage or high-risk TNBC, although I-SPY2 enrolled a broader high-risk stage II/III ERBB2-negative population and provided TNBC-specific data for the pCR analysis. The immune checkpoint inhibitors evaluated were pembrolizumab, atezolizumab, and durvalumab, representing both anti-PD-1 and anti-PD-L1 agents. Most trials evaluated immunotherapy in combination with neoadjuvant chemotherapy, whereas KEYNOTE-522 and IMpassion031 also included postoperative immunotherapy. Chemotherapy backbones differed across trials and included taxane-, anthracycline-, carboplatin-, and nab-paclitaxel-containing regimens. Differences were also present in disease stage, nodal-status reporting, PD-L1 assessment, pCR definition, immunotherapy schedule, and duration of follow-up (**Table 1**).

**Table 1:** Expanded characteristics of included studies.

Study	Population used	Stage	Nodal status	PD-L1 assay/cutoff	pCR definition	ICI drug/class	ICI timing/cycles	Follow-up
NeoTRIP Michelangelo	Early high-risk or locally advanced TNBC	Early high-risk/locally advanced; inflammatory disease included	NR	VENTANA SP142; IC ≥1% defined as PD-L1-positive	Absence of invasive cancer cells in breast and lymph nodes	Atezolizumab/anti-PD-L1	Atezolizumab 1200 mg q3w + carboplatin/nab-paclitaxel for 8 neoadjuvant cycles; anthracycline postoperatively	Primary pCR report; later follow-up approximately 54 months
KEYNOTE-522	Newly diagnosed, previously untreated early TNBC	Stage II–III	Stratified; node-positive and node-negative subgroups reported	PD-L1 IHC 22C3 pharmDx; CPS ≥1 positive	ypT0/Tis ypN0	Pembrolizumab/anti-PD-1	Pembrolizumab 200 mg q3w with paclitaxel/carboplatin ×4 cycles, then anthracycline/cyclophosphamide ×4 cycles; adjuvant pembrolizumab/placebo up to 9 cycles	Median 15.5 months in initial pCR analysis; later 36-month and OS follow-up reported
I-SPY2	High-risk stage II/III ERBB2-negative breast cancer; TNBC signature used for pCR	Stage II–III	Palpable nodes reported in ERBB2-negative population; TNBC-specific nodal data NR	PD-L1 not used for eligibility or stratification; assay/cutoff NR	Absence of invasive tumor in breast and regional nodes	Pembrolizumab/anti-PD-1	Pembrolizumab 200 mg q3w ×4 with weekly paclitaxel, followed by AC	Median follow-up 2.8 years for pembrolizumab pCR patients
IMpassion031	Previously untreated early-stage TNBC	Stage II–III; stage II 126/165 vs 129/168; stage III 38/165 vs 39/168	N0 109/165 vs 96/168; N1–3 56/165 vs 72/168	VENTANA SP142; IC ≥1% positive, IC <1% negative	ypT0/is ypN0	Atezolizumab/anti-PD-L1	Atezolizumab 840 mg q2w + nab-paclitaxel for 12 weeks, then dose-dense doxorubicin/cyclophosphamide for 8 weeks; adjuvant atezolizumab 1200 mg q3w ×11	Median 20.6 vs 19.8 months
GeparNuevo	Centrally confirmed early TNBC	cT1b–cT4a-d; stage ≥IIA in 113/174 (65%)	Nodal subgroup data available for pCR analysis; baseline arm-level nodal distribution not fully reported in main table	PD-L1-positive tumors 138/158 (87%); TC/IC scoring reported; assay/cutoff NR	ypT0 ypN0	Durvalumab/anti-PD-L1	Durvalumab/placebo q4w with optional 2-week window phase, then nab-paclitaxel followed by dose-dense EC	Primary pCR report; later long-term analysis median follow-up 86.4 months

AC, doxorubicin plus cyclophosphamide; CPS, combined positive score; EC, epirubicin plus cyclophosphamide; ERBB2, erb-b2 receptor tyrosine kinase 2; IC, tumor-infiltrating immune cells; ICI, immune checkpoint inhibitor; IHC, immunohistochemistry; NR, not reported; OS, overall survival; pCR, pathological complete response; PD-1, programmed cell death protein 1; PD-L1, programmed death-ligand 1; q2w, every 2 weeks; q3w, every 3 weeks; q4w, every 4 weeks; TC, tumor cells; TNBC, triple-negative breast cancer.

Staging definitions: cT1b–cT4a–d, clinical tumor stages T1b through T4a–d; N0, no regional lymph node metastasis; N1–3, regional lymph node involvement classified as N1 to N3; ypT0/is ypN0, no residual invasive cancer in the breast, with residual carcinoma *in situ* permitted, and no regional lymph node metastasis; ypT0 ypN0, no residual invasive or *in situ* cancer in the breast and no regional lymph node metastasis.

### 3.3 Risk of bias assessment

Using the original Cochrane Risk of Bias tool (RoB 1), most assessed domains were judged to be at low risk of bias. High risk was identified for allocation concealment in NeoTRIP Michelangelo, blinding of participants and personnel in I-SPY2, and incomplete outcome data in GeparNuevo. The remaining assessed domains, including random sequence generation, blinding of outcome assessment, selective reporting, and other bias, were judged to be at low risk in the respective trials. The domain-level judgments were consistent between the graphical assessment and the supplementary risk-of-bias table (**Figure 2**; **Supplementary Table 2**).

**Figure 2:**
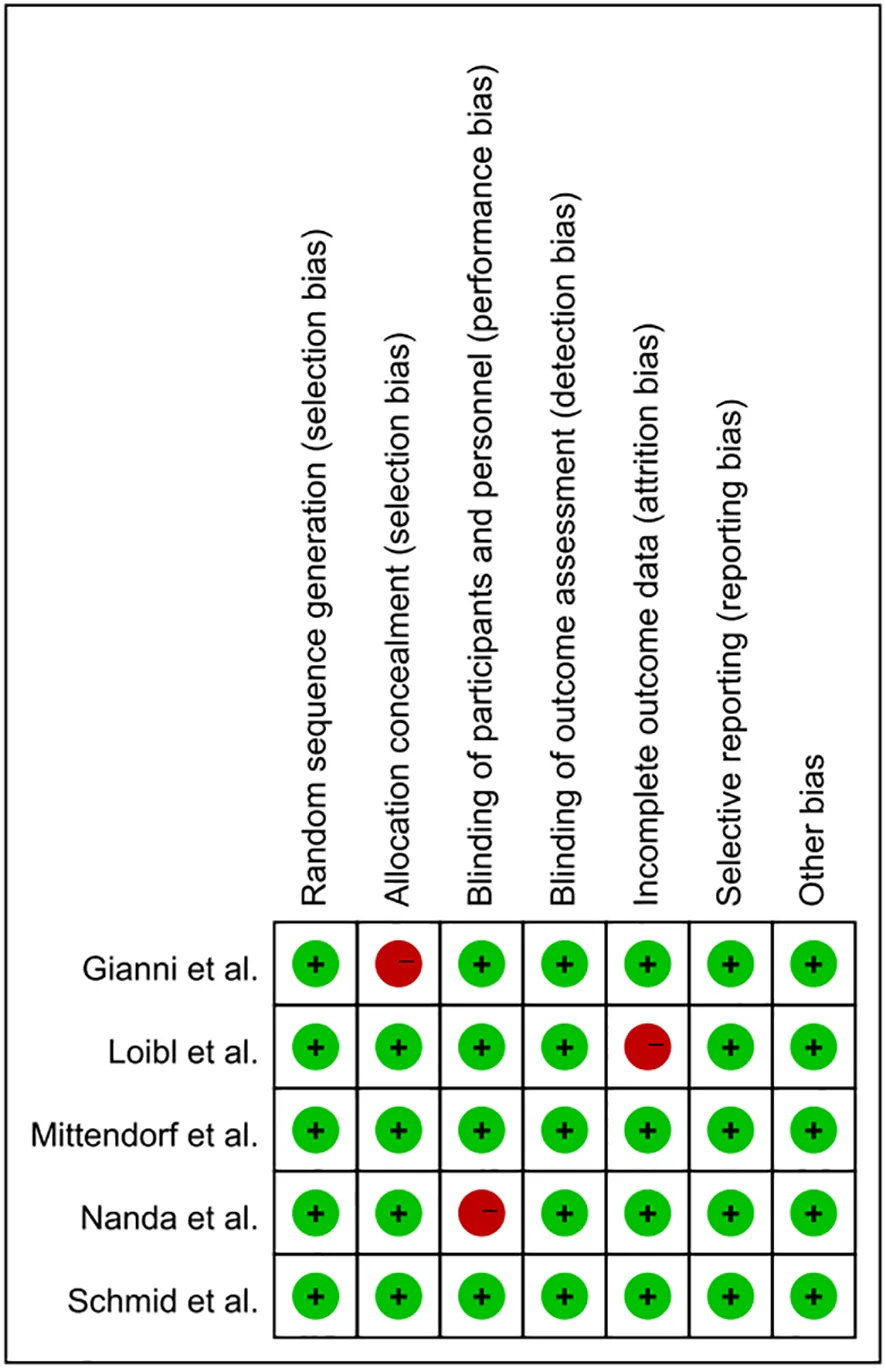
Risk-of-bias assessment of the included randomized controlled trials using the original Cochrane Risk of Bias tool (RoB 1). Green circles indicate low risk of bias and red circles indicate high risk of bias.

### 3.4 Pathological complete response

All five trials were included in the primary pCR analysis. Substantial between-study heterogeneity was observed (I² = 83.7%, P < 0.001), and a random-effects model was applied. The pooled estimate was higher for immunotherapy plus chemotherapy than for the control regimens (RR = 1.44, 95% CI 1.09–1.79) (**Figure 3**).

**Figure 3:**
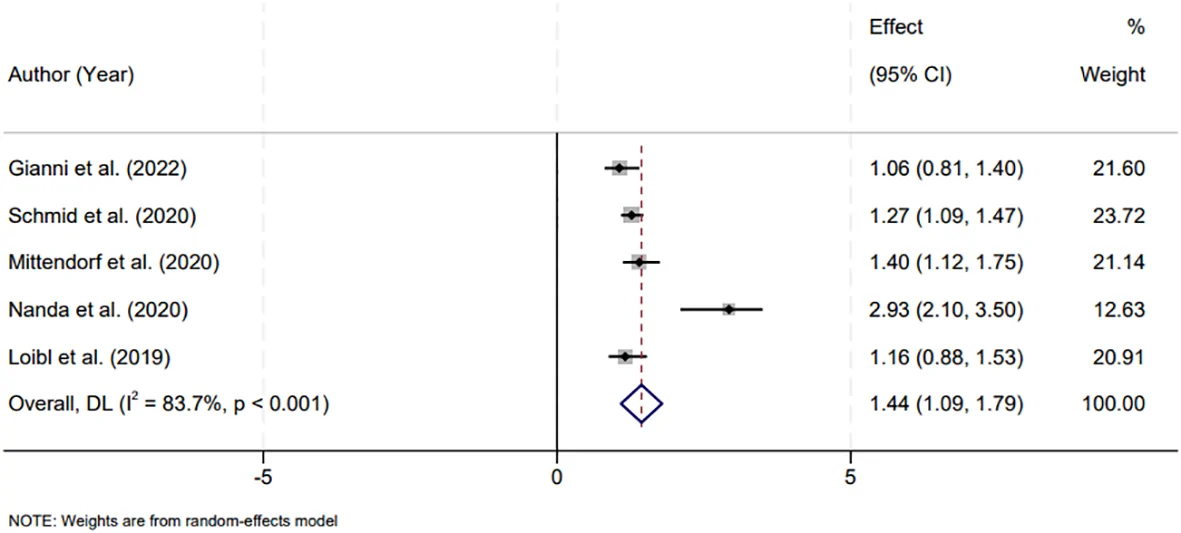
Forest plot showing the pooled effect of immunotherapy combined with chemotherapy versus control treatment on pathological complete response in patients with early-stage triple-negative breast cancer.

Although all trials defined pCR by the absence of invasive disease in the breast and regional lymph nodes, definitions differed in whether residual ductal carcinoma *in situ* was permitted, and analysis populations were not completely uniform. GeparNuevo used the stricter ypT0 ypN0 definition, whereas residual *in situ* disease was permitted in KEYNOTE-522 and IMpassion031 and was not explicitly specified in NeoTRIP Michelangelo or I-SPY2. For I-SPY2, observed TNBC-specific pCR data were used in the meta-analysis, while Bayesian model-estimated rates were retained descriptively. These methodological differences may have contributed to the observed heterogeneity (**Supplementary Table 3**).

Leave-one-out sensitivity analyses showed no material change in the direction of the pooled estimate after exclusion of individual studies (**Figure 4**).

**Figure 4:**
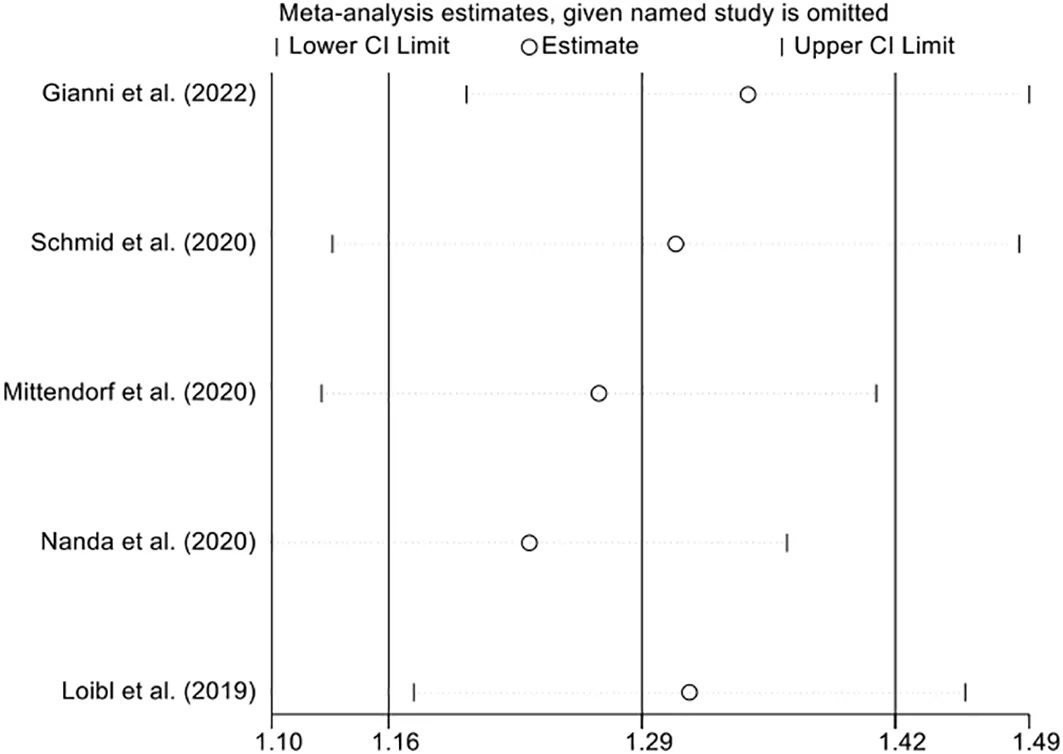
Sensitivity analysis of pathological complete response performed by sequential omission of each included study to evaluate the robustness of the pooled result.

### 3.5 Exploratory subgroup analyses of pathological complete response

Exploratory subgroup analyses were performed according to PD-L1 expression and lymph node status. The pooled estimate was higher with immunotherapy plus chemotherapy in the PD-L1-positive subgroup (RR = 1.36, 95% CI 1.18–1.57), whereas the estimate in the PD-L1-negative subgroup was less precise and crossed the null value (RR = 1.18, 95% CI 0.96–1.45). Corresponding estimates were 1.42 (95% CI 1.19–1.69) for node-positive disease and 1.21 (95% CI 1.02–1.43) for node-negative disease. These findings were considered exploratory and were not interpreted as evidence of differential effects between subgroups (**Table 2**).

**Table 2:** Exploratory subgroup analyses of pCR according to PD-L1 expression and lymph node status.

Subgroup	No. of studies	Contributing trials	Model	RR (95% CI)	P value	I² (%)	P for heterogeneity
PD-L1-positive	4	NeoTRIP Michelangelo; KEYNOTE-522; IMpassion031; GeparNuevo	Random-effects	1.36 (1.18–1.57)	<0.001	41.2	0.163
PD-L1-negative	4	NeoTRIP Michelangelo; KEYNOTE-522; IMpassion031; GeparNuevo	Random-effects	1.18 (0.96–1.45)	0.114	35.7	0.211
Lymph node-positive	3	KEYNOTE-522; IMpassion031; GeparNuevo	Random-effects	1.42 (1.19–1.69)	<0.001	46.5	0.112
Lymph node-negative	3	KEYNOTE-522; IMpassion031; GeparNuevo	Random-effects	1.21 (1.02–1.43)	0.027	28.9	0.228

CI, confidence interval; I², I-squared statistic; pCR, pathological complete response; PD-L1, programmed death-ligand 1; RR, risk ratio.

PD-L1 assessment varied across trials, including SP142-based immune-cell scoring and 22C3-based combined positive scoring, with different positivity thresholds. Detailed assay information was not uniformly available for all studies. Checkpoint-inhibitor class was therefore examined descriptively only; two trials evaluated anti-PD-1 therapy and three evaluated anti-PD-L1 therapy, and no formal between-class interaction test was performed because of the limited number of studies and between-trial heterogeneity (**Supplementary Table 4**).

### 3.6 Safety outcomes

Three trials contributed to the pooled analysis of grade 3–4 adverse events (AEs), yielding an RR of 1.18 (95% CI 1.05–1.33; I² = 52.6%). Four trials contributed data on serious AEs (RR = 1.42, 95% CI 1.12–1.79; I² = 21.4%) and AE-related treatment discontinuation (RR = 1.67, 95% CI 1.18–2.36; I² = 34.8%) (**Table 3**).

**Table 3:** Meta-analysis of safety outcomes.

Outcome	No. of studies	Contributing trials	Model	RR (95% CI)	P value	I² (%)	P for heterogeneity
Grade 3–4 AEs	3	NeoTRIP Michelangelo; KEYNOTE-522; IMpassion031	Random-effects	1.18 (1.05–1.33)	0.006	52.6	0.078
Serious AEs	4	NeoTRIP Michelangelo; KEYNOTE-522; IMpassion031; GeparNuevo	Fixed-effects	1.42 (1.12–1.79)	0.004	21.4	0.281
Treatment discontinuation due to AEs	4	NeoTRIP Michelangelo; KEYNOTE-522; IMpassion031; GeparNuevo	Fixed-effects	1.67 (1.18–2.36)	0.004	34.8	0.204

AE, adverse event; CI, confidence interval; I², I-squared statistic; RR, risk ratio.

Across trials, commonly reported grade 3–4 events included neutropenia, anemia, febrile neutropenia, leukopenia, and liver transaminase abnormalities. Definitions of AE-related treatment discontinuation were not fully uniform, and I-SPY2 was excluded from the pooled TNBC safety analyses because its safety data were reported primarily for the broader ERBB2-negative population rather than the TNBC subgroup (**Supplementary Table 5**).

Immune-related toxicity was reported using heterogeneous terminology and definitions, including immune-related AEs, immune-mediated AEs, and adverse events of special interest. Reported events included thyroid dysfunction, adrenal insufficiency, pneumonitis, hepatitis or liver enzyme abnormalities, colitis, skin toxicity, and infusion reactions. Because definitions, denominators, and reporting formats differed across trials, these outcomes were summarized descriptively rather than quantitatively pooled (**Supplementary Table 6**).

### 3.7 Long-term efficacy outcomes

Long-term outcomes were reported with different endpoints and follow-up durations. KEYNOTE-522 subsequently reported an event-free survival hazard ratio of 0.63 (95% CI 0.48–0.82), whereas IMpassion031 reported an event-free survival hazard ratio of 0.76 (95% CI 0.40–1.44) and a disease-free survival hazard ratio of 0.74 (95% CI 0.32–1.70). The later NeoTRIP Michelangelo report described approximately 5-year event-free survival rates of 70.6% and 74.9%, although an extractable hazard ratio was not available in the dataset used for this review. GeparNuevo reported longer-term invasive disease-free, distant disease-free, and overall survival estimates after a median follow-up of 86.4 months, whereas the I-SPY2 survival data were limited and exploratory. Because endpoint definitions, follow-up duration, data maturity, and availability of hazard ratios and confidence intervals differed substantially across trials, these outcomes were summarized descriptively and were not quantitatively pooled (**Supplementary Table 7**).

### 3.8 Assessment of publication bias

Formal assessment of publication bias was not performed because only five studies were included in the primary meta-analysis. With this number of studies, funnel-plot asymmetry and regression-based tests have limited power and would not provide a reliable assessment of small-study effects or publication bias.

## 4 Discussion

This systematic review and meta-analysis provides an updated synthesis of randomized evidence evaluating immune checkpoint inhibitors combined with chemotherapy in early-stage or high-risk triple-negative breast cancer. Unlike previous reviews that primarily emphasized overall efficacy or broadly pooled immune checkpoint blockade in early breast cancer, the present study integrates pCR, clinically relevant safety outcomes, and available long-term efficacy data specifically in early-stage TNBC. Its added value lies in examining pCR together with important sources of clinical and methodological heterogeneity, including pCR definitions, PD-L1 assessment, nodal status, immune checkpoint inhibitor class, treatment schedule, and analysis population. In addition, grade 3–4 adverse events, serious adverse events, treatment discontinuation, and immune-related toxicity were evaluated separately rather than as a single composite safety outcome. By distinguishing short-term pathological response from available long-term outcomes, this review provides a more balanced framework for interpreting the efficacy and safety of chemoimmunotherapy in early-stage TNBC (21–23).

The pooled pCR result showed a higher pathological response rate with immune checkpoint inhibitor plus chemotherapy than with the corresponding control regimens in early-stage or high-risk TNBC. This finding is biologically plausible given the immunogenic characteristics of TNBC, which frequently include increased tumor-infiltrating lymphocytes, relatively high genomic instability, and an immune-active tumor microenvironment. Cytotoxic chemotherapy may further enhance antitumor immune activity through tumor antigen release, immunogenic cell death, and modulation of immune effector and suppressor cell populations, thereby providing a biological rationale for combining chemotherapy with immune checkpoint blockade (24, 25). At the same time, the substantial heterogeneity observed in the primary pCR analysis indicates that the magnitude of the pooled association varied across trials. Several clinically relevant differences may have contributed to this variation, including chemotherapy backbone, use of carboplatin or nab-paclitaxel, sequencing and duration of immunotherapy, incorporation of postoperative immunotherapy, trial phase, and analysis population (26, 27). Differences in pCR definition may also have influenced the observed treatment estimates. KEYNOTE-522 and IMpassion031 permitted residual ductal carcinoma *in situ*, whereas GeparNuevo used the stricter ypT0 ypN0 definition; residual *in situ* disease was not explicitly specified in NeoTRIP Michelangelo or I-SPY2. Such variation in endpoint definition may alter the absolute proportion of patients classified as achieving pCR and therefore contribute to differences in study-level effect estimates. The exploratory subgroup analyses further suggested variation according to PD-L1 expression and lymph node status. The pooled estimate in the PD-L1-positive subgroup excluded the null value, whereas the estimate in the PD-L1-negative subgroup was less precise and crossed the null value. Similarly, the point estimate was numerically larger in node-positive than in node-negative disease. These patterns may reflect differences in baseline tumor burden, immune activity, disease biology, and trial composition, but they should be interpreted as descriptive subgroup patterns rather than definitive evidence of treatment-effect modification (28, 29).

The safety findings should be considered together with the observed pCR results when evaluating chemoimmunotherapy in early-stage TNBC. The pooled analyses showed higher risks of grade 3–4 adverse events, serious adverse events, and treatment discontinuation due to adverse events in the immunotherapy-containing groups. Commonly reported grade 3–4 toxicities included neutropenia, anemia, febrile neutropenia, leukopenia, and elevations in liver transaminases, reflecting the combined toxicity burden of multiagent chemotherapy and immune checkpoint blockade (30, 31). These events are clinically relevant in the neoadjuvant setting because treatment intensity, hematologic recovery, infection risk, hepatic function, and the timing of surgery must all be considered during therapy. In addition to conventional chemotherapy-associated toxicities, immune-related or immune-mediated adverse events represent a distinct component of treatment burden. Reported events included thyroid dysfunction, adrenal insufficiency, pneumonitis, hepatitis or liver enzyme abnormalities, colitis, skin reactions, and infusion-related reactions (32, 33). Their clinical management may differ substantially from that of standard cytotoxic toxicity and may require corticosteroids, endocrine replacement, specialist assessment, or prolonged follow-up. These considerations are particularly relevant in potentially curable early-stage disease, in which the therapeutic goal extends beyond tumor response to completion of multimodality treatment with acceptable toxicity. The available long-term efficacy findings also provide additional clinical context for the pCR results. KEYNOTE-522 subsequently reported favorable event-free survival findings, whereas IMpassion031, NeoTRIP Michelangelo, GeparNuevo, and I-SPY2 reported long-term outcomes with differing endpoints and follow-up durations. Taken together, the emerging evidence supports assessment of chemoimmunotherapy through multiple dimensions, including pathological response, treatment completion, serious toxicity, immune-mediated adverse events, and subsequent disease-control outcomes, rather than relying on pCR alone as the sole measure of clinical value.

The PD-L1 subgroup findings should be interpreted cautiously and as hypothesis-generating. In the present analysis, the confidence interval for the pooled estimate excluded the null value in the PD-L1-positive subgroup but crossed the null value in the PD-L1-negative subgroup. However, this difference in statistical significance does not establish a differential treatment effect between PD-L1 subgroups, particularly in the absence of a formal interaction test. Previous evidence has also suggested that PD-L1 may not function as a definitive predictive biomarker for chemoimmunotherapy in early-stage TNBC. In KEYNOTE-522, pembrolizumab-based therapy showed clinical activity across PD-L1-defined populations, while other studies have suggested that PD-L1 expression may also reflect an intrinsically more immune-active tumor microenvironment rather than a treatment-specific predictive effect (33–35). Interpretation is further complicated by substantial methodological variability in PD-L1 assessment. The included trials used different assays and scoring approaches, including VENTANA SP142 immune-cell scoring with an IC ≥1% cutoff and PD-L1 IHC 22C3 pharmDx with a combined positive score cutoff of CPS ≥1, while assay and cutoff information was incomplete in some studies. These methods are not directly interchangeable and may classify different patient populations as PD-L1 positive. Furthermore, only two trials evaluated anti-PD-1 agents and three evaluated anti-PD-L1 agents; given the limited number of studies and differences in study populations, chemotherapy backbones, treatment schedules, and PD-L1 assessment, no reliable formal comparison between checkpoint-inhibitor classes could be performed. Accordingly, the present subgroup findings do not support the use of a specific PD-L1 assay, cutoff, or checkpoint-inhibitor class for patient selection.

## 5 Limitations and future directions

Several limitations should be considered. First, only five randomized trials were included, limiting the statistical power of subgroup analyses, comparisons between anti-PD-1 and anti-PD-L1 agents, and reliable assessment of publication bias; therefore, biomarker- and class-based findings should be considered exploratory. Second, substantial heterogeneity in the primary pCR analysis may reflect differences in trial design, chemotherapy backbone, immunotherapy schedule, analysis population, disease-risk profile, and pCR definition, particularly whether residual ductal carcinoma *in situ* was permitted. Third, most subgroup analyses were based on trial-level rather than individual patient-level data, and heterogeneity in PD-L1 assays, scoring systems, and cutoffs further limited biomarker interpretation. Selected risk-of-bias concerns involving allocation concealment, blinding, and incomplete outcome data should also be acknowledged. Fourth, safety reporting was not fully standardized, particularly for immune-related adverse events and treatment discontinuation, precluding quantitative pooling of immune-related toxicity. Long-term efficacy outcomes could likewise not be pooled because of differences in endpoint definitions, follow-up duration, data maturity, and availability of effect estimates. Finally, although pCR is clinically meaningful in neoadjuvant triple-negative breast cancer, it remains an imperfect surrogate for long-term benefit. Future studies should standardize pCR, safety, and survival endpoints; harmonize PD-L1 assessment; and provide mature event-free and overall survival data. Larger randomized datasets, individual patient-level analyses, and prospectively defined multimarker studies may further clarify treatment-effect heterogeneity and support individualized treatment selection. Despite these limitations, this review provides an updated and integrated synthesis of randomized evidence on pathological response, safety, biomarker-related heterogeneity, and available long-term outcomes.

## 6 Conclusions

Overall, immune checkpoint inhibitors combined with chemotherapy were associated with a higher pCR rate in patients with early-stage or high-risk triple-negative breast cancer, although this was accompanied by increased risks of grade 3–4 adverse events, serious adverse events, and treatment discontinuation due to adverse events. These findings support improved pathological response with chemoimmunotherapy while underscoring the need to balance efficacy against treatment-related toxicity. Exploratory analyses showed variation in pooled estimates according to PD-L1 expression and nodal status, providing hypotheses for future biomarker-guided research. Further studies with standardized biomarker assessment and mature long-term follow-up are needed to determine whether improvements in pathological response translate into durable clinical benefit.

## Data Availability

The raw data supporting the conclusions of this article will be made available by the authors, without undue reservation.
