# Supplementary material for: Efficacy and safety of immune checkpoint inhibitors plus chemotherapy in early-stage triple-negative breast cancer: a systematic review and meta-analysis
Source: Front Oncol. 2026 Sep 24;16:1853057. doi: 10.3389/fonc.2026.1853057 (PMC13645687; doi:10.3389/fonc.2026.1853057)
Supplement: Supplementary file 1 [file Table1.docx]

Supplementary Table S1. Detailed search strategies for each database

| Database | Search strategy |
| --- | --- |
| PubMed | #1 “Triple Negative Breast Neoplasms”[Mesh] OR “triple-negative breast cancer”[Title/Abstract] OR “triple negative breast cancer”[Title/Abstract] OR TNBC[Title/Abstract] OR “triple-negative breast neoplasm*”[Title/Abstract] OR “triple negative breast neoplasm*”[Title/Abstract] #2 “Breast Neoplasms”[Mesh] OR breast cancer[Title/Abstract] OR breast neoplasm*[Title/Abstract] OR breast carcinoma*[Title/Abstract] #3 “early-stage”[Title/Abstract] OR “early stage”[Title/Abstract] OR “early-stage breast cancer”[Title/Abstract] OR “early breast cancer”[Title/Abstract] OR resectable[Title/Abstract] OR operable[Title/Abstract] OR neoadjuvant[Title/Abstract] OR adjuvant[Title/Abstract] #4 “Immunotherapy”[Mesh] OR immunotherapy[Title/Abstract] OR “immune checkpoint inhibitor*”[Title/Abstract] OR “checkpoint inhibitor*”[Title/Abstract] OR “immune checkpoint blockade”[Title/Abstract] OR PD-1[Title/Abstract] OR PD-L1[Title/Abstract] OR “programmed cell death protein 1”[Title/Abstract] OR “programmed death-ligand 1”[Title/Abstract] OR pembrolizumab[Title/Abstract] OR atezolizumab[Title/Abstract] OR nivolumab[Title/Abstract] OR durvalumab[Title/Abstract] OR dostarlimab[Title/Abstract] #5 “Antineoplastic Combined Chemotherapy Protocols”[Mesh] OR “Drug Therapy, Combination”[Mesh] OR chemotherapy[Title/Abstract] OR “combined chemotherapy”[Title/Abstract] OR chemoimmunotherapy[Title/Abstract] OR “immunotherapy combined with chemotherapy”[Title/Abstract] OR “chemotherapy plus immunotherapy”[Title/Abstract] #6 randomized controlled trial[Publication Type] OR controlled clinical trial[Publication Type] OR randomized[Title/Abstract] OR randomised[Title/Abstract] OR randomly[Title/Abstract] OR trial[Title/Abstract] OR placebo[Title/Abstract] Final search: ((#1 OR (#2 AND #3)) AND #4 AND #5 AND #6) |
| Embase | #1 'triple negative breast cancer'/exp OR 'triple negative breast cancer':ti,ab,kw OR 'triple-negative breast cancer':ti,ab,kw OR TNBC:ti,ab,kw OR 'triple negative breast neoplasm*':ti,ab,kw OR 'triple-negative breast neoplasm*':ti,ab,kw #2 'breast cancer'/exp OR 'breast tumor'/exp OR 'breast carcinoma':ti,ab,kw OR 'breast neoplasm*':ti,ab,kw OR 'breast cancer':ti,ab,kw #3 'early stage':ti,ab,kw OR 'early-stage':ti,ab,kw OR 'early breast cancer':ti,ab,kw OR resectable:ti,ab,kw OR operable:ti,ab,kw OR neoadjuvant:ti,ab,kw OR adjuvant:ti,ab,kw #4 'cancer immunotherapy'/exp OR 'immunotherapy'/exp OR 'immune checkpoint inhibitor'/exp OR immunotherapy:ti,ab,kw OR 'immune checkpoint inhibitor*':ti,ab,kw OR 'checkpoint inhibitor*':ti,ab,kw OR 'immune checkpoint blockade':ti,ab,kw OR 'pd 1':ti,ab,kw OR 'pd l1':ti,ab,kw OR pembrolizumab:ti,ab,kw OR atezolizumab:ti,ab,kw OR nivolumab:ti,ab,kw OR durvalumab:ti,ab,kw OR dostarlimab:ti,ab,kw #5 'chemotherapy'/exp OR 'combined chemotherapy'/exp OR chemotherapy:ti,ab,kw OR chemoimmunotherapy:ti,ab,kw OR 'immunotherapy combined with chemotherapy':ti,ab,kw OR 'chemotherapy plus immunotherapy':ti,ab,kw #6 'randomized controlled trial'/exp OR 'clinical trial'/exp OR random*:ti,ab,kw OR placebo:ti,ab,kw OR trial:ti,ab,kw Final search: ((#1 OR (#2 AND #3)) AND #4 AND #5 AND #6) |
| Web of Science Core Collection | #1 TS=(“triple-negative breast cancer” OR “triple negative breast cancer” OR TNBC OR “triple-negative breast neoplasm*” OR “triple negative breast neoplasm*”) #2 TS=(“breast cancer” OR “breast neoplasm*” OR “breast carcinoma*”) #3 TS=(“early-stage” OR “early stage” OR “early breast cancer” OR resectable OR operable OR neoadjuvant OR adjuvant) #4 TS=(immunotherapy OR “immune checkpoint inhibitor*” OR “checkpoint inhibitor*” OR “immune checkpoint blockade” OR “PD-1” OR “PD-L1” OR pembrolizumab OR atezolizumab OR nivolumab OR durvalumab OR dostarlimab) #5 TS=(chemotherapy OR “combined chemotherapy” OR chemoimmunotherapy OR “immunotherapy combined with chemotherapy” OR “chemotherapy plus immunotherapy”) #6 TS=(randomized OR randomised OR randomly OR trial OR placebo OR “controlled trial” OR “randomized controlled trial”) Final search: ((#1 OR (#2 AND #3)) AND #4 AND #5 AND #6) |
| Cochrane Library | #1 MeSH descriptor: [Triple Negative Breast Neoplasms] explode all trees #2 (“triple-negative breast cancer” OR “triple negative breast cancer” OR TNBC OR “triple-negative breast neoplasm*” OR “triple negative breast neoplasm*”):ti,ab,kw #3 MeSH descriptor: [Breast Neoplasms] explode all trees #4 (“breast cancer” OR “breast neoplasm*” OR “breast carcinoma*”):ti,ab,kw #5 (“early-stage” OR “early stage” OR “early breast cancer” OR resectable OR operable OR neoadjuvant OR adjuvant):ti,ab,kw #6 MeSH descriptor: [Immunotherapy] explode all trees #7 (immunotherapy OR “immune checkpoint inhibitor*” OR “checkpoint inhibitor*” OR “immune checkpoint blockade” OR “PD-1” OR “PD-L1” OR pembrolizumab OR atezolizumab OR nivolumab OR durvalumab OR dostarlimab):ti,ab,kw #8 MeSH descriptor: [Antineoplastic Combined Chemotherapy Protocols] explode all trees #9 (chemotherapy OR “combined chemotherapy” OR chemoimmunotherapy OR “immunotherapy combined with chemotherapy” OR “chemotherapy plus immunotherapy”):ti,ab,kw #10 (randomized OR randomised OR randomly OR trial OR placebo OR “controlled trial”):ti,ab,kw Final search: ((#1 OR #2 OR ((#3 OR #4) AND #5)) AND (#6 OR #7) AND (#8 OR #9) AND #10) |

Supplementary Table S2. Cochrane RoB 1 domain-level assessment

| Study | Random sequence generation | Allocation concealment | Blinding of participants/personnel | Blinding of outcome assessment | Incomplete outcome data | Selective reporting | Other bias |
| --- | --- | --- | --- | --- | --- | --- | --- |
| NeoTRIP Michelangelo | Low risk | High risk | Low risk | Low risk | Low risk | Low risk | Low risk |
| KEYNOTE-522 | Low risk | Low risk | Low risk | Low risk | Low risk | Low risk | Low risk |
| I-SPY2 | Low risk | Low risk | High risk | Low risk | Low risk | Low risk | Low risk |
| IMpassion031 | Low risk | Low risk | Low risk | Low risk | Low risk | Low risk | Low risk |
| GeparNuevo | Low risk | Low risk | Low risk | Low risk | High risk | Low risk | Low risk |

Abbreviation: RoB 1, Cochrane Risk of Bias tool, version 1.

Supplementary Table S3. pCR definitions and analysis populations

| Study | Exact pCR definition | Notation | Residual DCIS permitted in pCR definition | ICI pCR data used in main pCR meta-analysis | Control pCR data used in main pCR meta-analysis | Additional trial-reported pCR information | Analysis population | Arm-level RR (95% CI) |
| --- | --- | --- | --- | --- | --- | --- | --- | --- |
| NeoTRIP Michelangelo | Absence of invasive cancer cells in breast and lymph nodes | No invasive disease in breast/nodes; exact yp notation not explicitly reported in the main abstract | Not explicitly reported | 67/138 (48.6%) | 63/142 (44.4%) | — | ITT; randomized 138 vs 142 | 1.09 (0.85–1.41) |
| KEYNOTE-522 | No residual invasive cancer in breast and sampled regional lymph nodes | ypT0/Tis ypN0 | Yes | 260/401 (64.8%) | 103/201 (51.2%) | — | First pCR interim ITT analysis; overall randomized population 784 vs 390 | 1.27 (1.09–1.47) |
| I-SPY2 | Absence of invasive tumor in breast and regional nodes at surgery | No invasive disease in breast/nodes | Not explicitly reported | 19/28 (67.9%) | 18/79 (22.8%) | Bayesian model-estimated pCR: 60% vs 22%, reported descriptively | TNBC signature; pembrolizumab n=29, control n=85; modified ITT/evaluable reporting | 2.98 (1.84–4.81) |
| IMpassion031 | No residual invasive disease in breast and lymph nodes | ypT0/is ypN0 | Yes | 95/165 (57.6%) | 69/168 (41.1%) | — | ITT 165 vs 168 | 1.40 (1.12–1.75) |
| GeparNuevo | No microscopic evidence of residual invasive and non-invasive viable tumor cells in all resected breast and axillary specimens | ypT0 ypN0 | No | 47/88 (53.4%) | 38/86 (44.2%) | — | Randomized/mITT 88 vs 86 | 1.21 (0.89–1.64) |

Abbreviations: CI, confidence interval; DCIS, ductal carcinoma in situ; ICI, immune checkpoint inhibitor; ITT, intention-to-treat; mITT, modified intention-to-treat; pCR, pathological complete response; RR, risk ratio; TNBC, triple-negative breast cancer.

Staging definitions: ypT0/Tis ypN0 (also written as ypT0/is ypN0), no residual invasive cancer in the breast, with residual carcinoma in situ permitted, and no regional lymph node metastasis; ypT0 ypN0, no residual invasive or in situ cancer in the breast and no regional lymph node metastasis.

Supplementary Table S4. PD-L1 assay characteristics, subgroup data source, and checkpoint inhibitor class

| Study | ICI drug | ICI class | PD-L1 assay/platform | Scoring system | Cutoff | Included in Table 2 PD-L1 subgroup analysis | PD-L1-positive subgroup data source | PD-L1-negative subgroup data source | Use/interpretation |
| --- | --- | --- | --- | --- | --- | --- | --- | --- | --- |
| NeoTRIP Michelangelo | Atezolizumab | PD-L1 inhibitor | VENTANA SP142 | IC | IC ≥1% positive; IC <1% negative | Yes | Study-reported PD-L1 subgroup results from the original trial report and available supplementary materials; arm-level n/N not consistently displayed in the main text | Study-reported PD-L1 subgroup results from the original trial report and available supplementary materials; arm-level n/N not consistently displayed in the main text | Exploratory PD-L1 subgroup; PD-L1 expression was associated with higher pCR, but there was no clear evidence of a predictive interaction for atezolizumab benefit |
| KEYNOTE-522 | Pembrolizumab | PD-1 inhibitor | PD-L1 IHC 22C3 pharmDx | CPS | CPS ≥1 positive; CPS <1 negative | Yes | 230/334 (68.9%) vs 90/164 (54.9%) | 29/64 (45.3%) vs 10/33 (30.3%) | Exploratory PD-L1 subgroup; pCR benefit was observed in both PD-L1-positive and PD-L1-negative subgroups |
| I-SPY2 | Pembrolizumab | PD-1 inhibitor | NR | NR | PD-L1 not used for eligibility or stratification | No | NR | NR | Not used for PD-L1 subgroup meta-analysis |
| IMpassion031 | Atezolizumab | PD-L1 inhibitor | VENTANA SP142 | IC | IC ≥1% positive; IC <1% negative | Yes | 53/77 (68.8%) vs 37/75 (49.3%) | 42/88 (47.7%) vs 32/93 (34.4%) | Exploratory for comparison between PD-1 and PD-L1 inhibitor classes; the PD-L1-positive co-primary pCR analysis did not cross the prespecified significance boundary |
| GeparNuevo | Durvalumab | PD-L1 inhibitor | PD-L1 IHC; exact clone/platform NR | TC/IC | PD-L1-positive tumors 138/158 (87%); detailed cutoff NR | Yes | Study-reported PD-L1 subgroup results from the original trial report and available supplementary materials; detailed arm-level n/N not consistently displayed in the main text | Study-reported PD-L1 subgroup results from the original trial report and available supplementary materials; detailed arm-level n/N not consistently displayed in the main text | Exploratory descriptive PD-L1 subgroup; not powered for formal class comparison |

Comparisons according to checkpoint inhibitor class were descriptive and exploratory. No formal between-class interaction test was performed because only two anti-PD-1 and three anti-PD-L1 trials were available and substantial clinical and methodological heterogeneity existed across trials.

Abbreviations: CPS, combined positive score; IC, tumor-infiltrating immune cells; ICI, immune checkpoint inhibitor; IHC, immunohistochemistry; NR, not reported; pCR, pathological complete response; PD-1, programmed cell death protein 1; PD-L1, programmed death-ligand 1; TC, tumor cells.

Supplementary Table S5. Safety extraction data aligned with Table 3

| Study | Safety denominator ICI/control | Grade 3–4 AE extraction data, n/N | Common grade 3–4 AEs available from reports | SAE extraction data, n/N | AE-discontinuation extraction data, n/N | Discontinuation definition | Grade 3–4 AE analysis in Table 3 | SAE analysis in Table 3 | AE-discontinuation analysis in Table 3 |
| --- | --- | --- | --- | --- | --- | --- | --- | --- | --- |
| NeoTRIP Michelangelo | 138/140 | 94/138 vs 83/140 | Liver transaminase abnormalities more frequent with atezolizumab; event-level counts NR | 10/138 vs 6/140 | 13/138 vs 12/140 | AE leading to treatment discontinuation; exact component-level definition NR. | Yes | Yes | Yes |
| KEYNOTE-522 | 781/389 | 601/781 vs 269/389 | Neutropenia, decreased neutrophil count, anemia, febrile neutropenia, decreased white blood cell count | 129/781 vs 53/389 | 81/781 vs 16/389 | AE leading to discontinuation of any study intervention across neoadjuvant/adjuvant phases | Yes | Yes | Yes |
| I-SPY2 | 69/181 ERBB2-negative safety population | Not included | Febrile neutropenia 6/69 vs 13/181; anemia 5/69 vs 10/181; fatigue 5/69 vs 1/181; diarrhea 5/69 vs 4/181; nausea 4/69 vs 0/181 | NR | NR | NR | No | No | No |
| IMpassion031 | 164/167 | 113/164 vs 84/167 | Neutropenia, decreased neutrophil count, febrile neutropenia, anemia, leukopenia, hypertension, ALT/AST increase | 35/164 vs 16/167 | 7/164 vs 7/167 | AE leading to any treatment discontinuation | Yes | Yes | Yes |
| GeparNuevo | 92/82 | Not included | NR | 25/92 vs 15/82 | 16/92 vs 10/82 | AE leading to treatment discontinuation; exact component-level definition NR. | No | Yes | Yes |

Abbreviations: AE, adverse event; ALT, alanine aminotransferase; AST, aspartate aminotransferase; ERBB2, erb-b2 receptor tyrosine kinase 2; ICI, immune checkpoint inhibitor; NR, not reported; SAE, serious adverse event.

Supplementary Table S6. Immune-related adverse events / immune-mediated AEs / AESIs

| Study | Trial terminology | Any-grade n/N | Grade 3–4 n/N | Thyroid | Adrenal | Pneumonitis | Hepatitis | Colitis | Skin toxicity | Infusion reaction | Use in analysis |
| --- | --- | --- | --- | --- | --- | --- | --- | --- | --- | --- | --- |
| NeoTRIP Michelangelo | Immune-mediated AE / AESI terminology NR | NR | NR | NR | NR | NR | Transaminase/liver enzyme abnormalities reported more often with atezolizumab | NR | NR | NR | Descriptive only |
| KEYNOTE-522 | AEs of special interest / immune-mediated AEs and infusion reactions | 304/781 (38.9%) vs 71/389 (18.3%) | 101/781 (12.9%) vs 7/389 (1.8%) | Hypothyroidism 118/781 (15.1%) vs 22/389 (5.7%); hyperthyroidism 41/781 (5.2%) vs 7/389 (1.8%); thyroiditis 16/781 (2.0%) vs NR | Adrenal insufficiency 20/781 (2.6%) vs 0/389 | Pneumonitis 17/781 (2.2%) vs 6/389 (1.5%) | Immune-mediated hepatitis/ALT increase reported | Colitis 13/781 (1.7%) vs 3/389 (0.8%) | Severe skin reaction 45/781 (5.7%) vs 4/389 (1.0%) | Infusion-related reaction 141/781 (18.0%) vs 45/389 (11.6%) | Descriptive only |
| I-SPY2 | Selected irAEs | Unique total NR | Event-level total NR | Thyroid abnormalities 9/69 (13.0%) vs 0/181 | Adrenal insufficiency 6/69 (8.7%) vs 0/181 | 3/69 vs 2/181; grade 3–4: 0 vs 1/181 | 2/69 vs 0/181; grade 3–4: 2/69 vs 0 | 1/69 vs 1/181; grade 3–4: 1/69 vs 1/181 | Pruritus 22/69 vs 22/181; grade 3–4: 0 vs 1/181 | NR | Descriptive only |
| IMpassion031 | AESI | 115/164 (70.1%) vs 101/167 (60.5%) | 24/164 (14.6%) vs 20/167 (12.0%) | Hypothyroidism 11/164 vs 2/167; hyperthyroidism 5/164 vs 0/167 | 0/164 vs 1/167 | 2/164 vs 2/167; grade 3–4: 1/164 vs 0/167 | 2/164 vs 1/167; grade 3–4: 0 vs 0 | 1/164 vs 1/167; grade 3–4: 1/164 vs 0/167 | Rash 80/164 vs 82/167; grade 3–4: 6/164 vs 6/167 | 17/164 vs 11/167; grade 3–4: 1/164 vs 1/167 | Descriptive only |
| GeparNuevo | Immune-related AE | Unique total NR | NR | Thyroid dysfunction any grade 47% | NR | NR | NR | NR | NR | NR | Descriptive only |

Abbreviations: AE, adverse event; AESI, adverse event of special interest; ALT, alanine aminotransferase; irAE, immune-related adverse event; NR, not reported.

Supplementary Table S7. Long-term efficacy outcomes

| Study | Follow-up duration | EFS HR | DFS/iDFS HR | DDFS HR | OS HR | Use in analysis | Notes |
| --- | --- | --- | --- | --- | --- | --- | --- |
| NeoTRIP Michelangelo | Primary pCR report immature; later update approximately 54 months | HR NR; 5-year EFS approx. 70.6% vs 74.9% | NR | NR | NR | Descriptive only | Endpoint definitions and follow-up differ from other trials |
| KEYNOTE-522 | 15.5 months in initial pCR report; later 36-month and final OS follow-up | 0.63 (0.48–0.82) in later EFS analysis | NR | NR | Later OS benefit reported in final OS analysis | Descriptive only | Survival endpoints were not pooled with pCR outcome |
| I-SPY2 | Median 2.8 years for pembrolizumab arm pCR patients | Formal HR NR | NR | NR | NR | Descriptive only | Small exploratory survival analysis |
| IMpassion031 | 20.6 vs 19.8 months | 0.76 (0.40–1.44) | DFS 0.74 (0.32–1.70) | NR | 0.69 (0.25–1.87) | Descriptive only | Immature secondary time-to-event endpoints |
| GeparNuevo | Long-term analysis median 86.4 months | NR | iDFS 0.56 | 0.41 | 0.33 | Descriptive only | Long-term endpoints differ from other trials |

Abbreviations: DDFS, distant disease-free survival; DFS, disease-free survival; EFS, event-free survival; HR, hazard ratio; iDFS, invasive disease-free survival; NR, not reported; OS, overall survival; pCR, pathological complete response.
